# Another Rubber Patent

**Published:** 1869-06

**Authors:** 


### ANOTHER RUBBER PATENT.

ON the 19th day of January, 1859, Letters Patent No. 85,927 was granted to Robert Haering, for "*An Improved Mode of Mounting Artificial Teeth.*

The description as given by Dr. Haering in his specifications are as follows, viz.:

My invention relates to an improvement in securing artificial teeth upon bases or plates of hard or vulcanized rubber or other gum.

In order to enable others skilled in the art to practice my invention, I will now proceed to describe the manner of carrying it into effect.

A plaster cast of the mouth to which the teeth are to be fitted, is first made. A coating of wax, corresponding in shape and thickness to the "base" required, is then applied to the cast and round the bases of the teeth, which are thus held in their proper position.

The teeth and base are now covered with plaster, which is applied in a soft state, but soon hardens, forming the second division of a mould, the other part of which is the cast first made.

After the portion of the mould last made has hardened, the two divisions are separated, the wax is removed, and prepared rubber is substituted for the same, the sections of the mould being brought together so as to force the gum into all the interstices formerly occupied by the wax.

The mould is then placed in an oven and subjected to heat for such a length of time as may be necessary to harden the gum.

When the operation is complete, the plaster is broken away and the teeth will be found securely "set" or "mounted" on a base of gum, which is hard, tough, corresponds in shape to the wax base first made, and possesses many advantages over bases made of ordinary materials.

Without here claiming broadly the application of hard rubber or gum as a base for artificial teeth,

I claim as my invention, and desire to secure by Letters Patent,—

Artificial teeth, having gum bases, to which the teeth are secured, as described; that is, by "setting" the teeth in prepared gum on a mould, embedding the whole in plaster, and hardening the gum by the application of heat.

In testimony whereof, I have signed my name to this specification, in the presence of two subscribing witnesses.

ROBERT HAERING.

*Witnesses:*

SAM JOHN STORRS,

JOHN A. FOSTER.

This patent as we understand it, covers the ground claimed by the patent of J. A. Cumings, and now held by the Dental Vulcanite Company. We are not advised as to priority; and as there has been no proceedings to determine this matter that we are aware of, we presume they must be twins. Both are calling lustily for recognition by, and support from the Dental profession, at the same time threatening all who dare infringe. We would advise the profession, in order to be perfectly secure to take out a license under each, and then all will be right till *another* patent is obtained for the same thing. Pile it on, gentlemen; the brethren of the Dental profession are long-suffering. We are in favor of Patents in our profession; if any man gets a patent, especially if it be for something already patented, a general exchange might be made and all come out square.

We have been asked many questions in reference to these patents, and all we have to say is, gentlemen, buy every patent that comes along, especially if it has any thing to do with rubber? T.

